# Rapid Screening Method for Detecting Ethinyl Estradiol in Natural Water Employing Voltammetry

**DOI:** 10.1155/2016/3217080

**Published:** 2016-09-21

**Authors:** Chalder Nogueira Nunes, Lucas Ely Pauluk, Maria Lurdes Felsner, Vanessa Egéa dos Anjos, Sueli Pércio Quináia

**Affiliations:** ^1^Departamento de Química, Universidade Estadual do Centro-Oeste, 85040-080 Guarapuava, PR, Brazil; ^2^Departamento de Química, Universidade Estadual de Ponta Grossa, 84030-900 Ponta Grossa, PR, Brazil

## Abstract

17*α*-Ethinyl estradiol (EE2), which is used worldwide in the treatment of some cancers and as a contraceptive, is often found in aquatic systems and is considered a pharmaceutically active compound (PhACs) in the environment. Current methods for the determination of this compound, such as chromatography, are expensive and lengthy and require large amounts of toxic organic solvents. In this work, a voltammetric procedure is developed and validated as a screening tool for detecting EE2 in water samples without prior extraction, clean-up, or derivatization steps. Application of the method we elaborate here to EE2 analysis is unprecedented. EE2 detection was carried out using differential pulse adsorptive cathodic stripping voltammetry (DP AdCSV) with a hanging mercury drop electrode (HMDE) in pH 7.0 Britton-Robinson buffer. The electrochemical process of EE2 reduction was investigated by cyclic voltammetry at different scan rates. Electroreduction of the hormone on a mercury electrode exhibited a peak at −1.16 ± 0.02 V versus Ag/AgCl. The experimental parameters were as follows: −0.7 V accumulation potential, 150 s accumulation time, and 60 mV s^−1^ scan rate. The limit of detection was 0.49 *μ*g L^−1^ for a preconcentration time of 150 s. Relative standard deviations were less than 13%. The method was applied to the detection of EE2 in water samples with recoveries ranging from 93.7 to 102.5%.

## 1. Introduction

**Figure 1:**
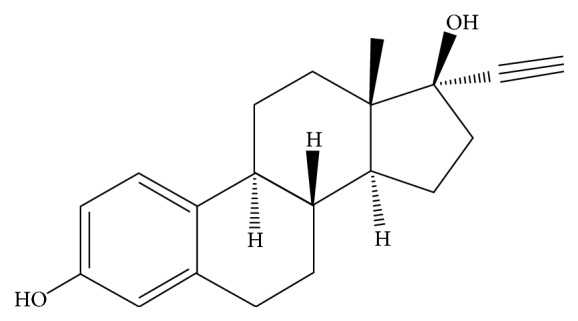
Chemical structure of EE2.

Ethinyl estradiol (EE2) or 17*α*-ethinyl estradiol (C_20_H_24_O_2_, Figure 1) is a synthetic hormone, which is a derivative of the natural hormone estradiol. EE2 is present in almost all formulations of oral contraceptive pills, as a contaminant in livestock manure used as fertilizer, and in aquaculture, where it is used to produce monosexual populations [1–4]. It is a pharmaceutically active compound (PhAC) that is introduced into aquatic environments through human and animal excretion [4]. The discharge of these compounds into natural waters occurs primarily via wastewater systems, because these chemicals are not completely eliminated by sewage treatment. EE2 is passed with urine into sewage in unmetabolized form and does not decompose [1, 2, 5].

Some PhACs have been detected in natural waters at concentrations associated with a wide range of adverse effects in nontarget organisms. EE2 is classified as an endocrine disruption agent (EDS), because it can mimic hormones or interfere with the action of endogenous hormones. Estrogens such as EE2 have been widely studied with regard to their high estrogen potential and effects on aquatic life [4, 6–8].

The determination of EE2 in environmental samples, pharmaceutical formulations, and biological samples has been performed by many analytical techniques including spectrometry, liquid and gas chromatography, electrophoresis, immunoassay, and electrochemistry [9–11]. Methods widely used to determine EE2 in environmental samples are based on chromatographic techniques such as high-performance liquid chromatography (HPLC), liquid chromatography (LC), and liquid chromatography-mass spectrometry (LC/MS) [5, 8]. These methods, which have high sensitivity, selectivity, and accuracy and a low limit of quantification (LOQ), are easily automated and can carry out the simultaneous determination of several targets [12]. However, they possess disadvantages that include the inability to perform in situ measurements, laborious procedures, expensive reagents and equipment, the need of skilled operators, and the frequent requirement of several sample preparation steps such as extraction, clean-up, and preconcentration [13, 14].

Electroanalytical techniques enable rapid analysis through advantages that include screening capability, high sensitivity, ease of operation, potential for miniaturization and in situ measurement, low cost, and freedom from sample treatment and preconcentration [12, 15]. Also, voltammetric techniques do not require the use of toxic organic solvents. Solid electrodes have produced satisfactory results for some samples [16]. However, these electrodes are more easily poisoned, do not exhibit reproducible surfaces, and require exhaustive cleaning, which are disadvantages relative to the hanging mercury drop electrode (HMDE) [12, 17]. Voltammetric methods have been used to determine organic compounds in various matrices such as biological materials and pharmaceutical formulations [10]. To date, no attempt has been made to determine EE2 in environmental samples using voltammetry at an HMDE.

In this paper, we describe an adsorptive stripping voltammetric procedure as a rapid screening tool for the determination of EE2 in natural water without a prior extraction or clean-up step. We present the results of an in-house validation study that assesses the parameters of selectivity, linearity, limit of detection (LOD), limit of quantification (LOQ), precision (repeatability and intermediate precision), and accuracy.

## 2. Materials and Methods

### 2.1. Chemicals and Apparatus

All reagents were of analytical grade and were used without further purification. Solutions were prepared with ultrapure water obtained from a TKA GenPure UV system (USA). Standard stock solutions of EE2 (Sigma Aldrich) were prepared in methanol (Sigma Aldrich). Britton-Robinson (B-R) universal buffers used as supporting electrolytes were prepared with 0.1 mol L^−1^ of sodium perchlorate, 0.04 mol L^−1^ of phosphoric acid, 0.04 mol L^−1^ of acetic acid, and 0.04 mol L^−1^ of boric acid. The pH of the buffer was adjusted with 2.0 mol L^−1^ NaOH or HCl. Solutions of commercial humic acid (HA, Sigma Aldrich) containing about 35% dissolved organic carbon (DOC) were prepared with ultrapure water. Water samples were collected from the rivers of Guarapuava, Brazil.

Voltammetric measurements were carried out with a Metrohm 757 VA analyzer controlled by a 757 VA Computrace computer program. Cyclic voltammetry and differential pulse adsorptive cathodic stripping voltammetry (DP AdCSV) were performed using a three-electrode system consisting of a Ag/AgCl reference electrode (3.0 mol L^−1^ KCl), a platinum wire auxiliary electrode, and a hanging mercury drop working electrode (HMDE). Ultrapure nitrogen was used as purge gas to remove oxygen during measurements. pH was measured with a combination Ag/AgCl-glass electrode and potentiometer (Hanna).

### 2.2. Optimization of the Analytical Procedure

Cyclic voltammetry (CV) was conducted to determine the mass transport behavior of EE2 (120 *μ*g L^−1^) in pH 6.3 B-R buffer at an HMDE. The CV parameters of accumulation time (*t*_ac_) and potential (*E*_ac_) were 150 s and −0.7 V, respectively, and the scan rate was 50 to 550 mV s^−1^.

The conditions for DP AdCSV were optimized in a cell containing 20.0 *μ*g L^−1^ EE2, 1.0 mL buffer solution (pH 7.0), and 9.0 mL ultrapure water. The following voltammetric parameters were evaluated over a pH range of 6.0–9.0: scan rate (45–105 mV s^−1^), accumulation potential (−0.5 to −0.9 V), accumulation time (60–450 s), pulse time (30–70 ms), pulse amplitude (50–100 mV), and equilibration time (0–7 s). Optimization of DP AdCSV parameters was based on the magnitude of the EE2 peak current (*I*_*p*_) and the resolution of the voltammetric signal.

### 2.3. In-House Validation of the Method

The standard addition method was used to quantitate EE2 by DP AdCSV in natural water to minimize the matrix effect. Linearity was evaluated over a range of 2.0 to 96.0 *μ*g L^−1^ EE2 (*t*_ac_ = 150 s). Measurements were conducted in triplicate. Linearity was checked by linear regression analysis with analysis of variance (ANOVA) and a lack-of-fit test at the 95% confidence level. Statistical analyses of the data were obtained with Minitab for Windows 16.2.2 software [18]. The Grubbs test was used to detect outliers at the 95% confidence level [19, 20].

The LOD and LOQ for determination of EE2 in natural water solutions were calculated from the peak current obtained from twenty replicates in supporting electrolyte (1 : 9 of B-R buffer and water) using the following equations: LOD = 3SD/*m* and LOQ = 10SD/*m*, where “SD” is the standard deviation of the peak current and “*m*” is the slope of the analytical curve [21].

The selectivity of the method was checked by analyzing standard solutions of bulk EE2 (9.5 *μ*g L^−1^) in the presence of humic acid (HA) at concentrations of 2.0 to 50.0 mg COD L^−1^ [12].

The precision of the method was evaluated in terms of repeatability and intermediate precision. Results were expressed as a relative standard deviation (RSD). The repeatability test consisted of five successive measurements of 10.1 *μ*g L^−1^ EE2 solutions prepared in quintuplicate over one day. Intermediate precision was calculated from repeated analyses of 10.1 *μ*g L^−1^ EE2 over five consecutive days [21]. The accuracy of the method was evaluated in terms of recovery from ultrapure water spiked with 8.0 and 10.0 *μ*g L^−1^ EE2 and natural water spiked with 16.0 *μ*g L^−1^ EE2 [12].

## 3. Results and Discussion

### 3.1. Electrochemical Behavior of Ethinyl Estradiol at HMDE

**Figure 2:**
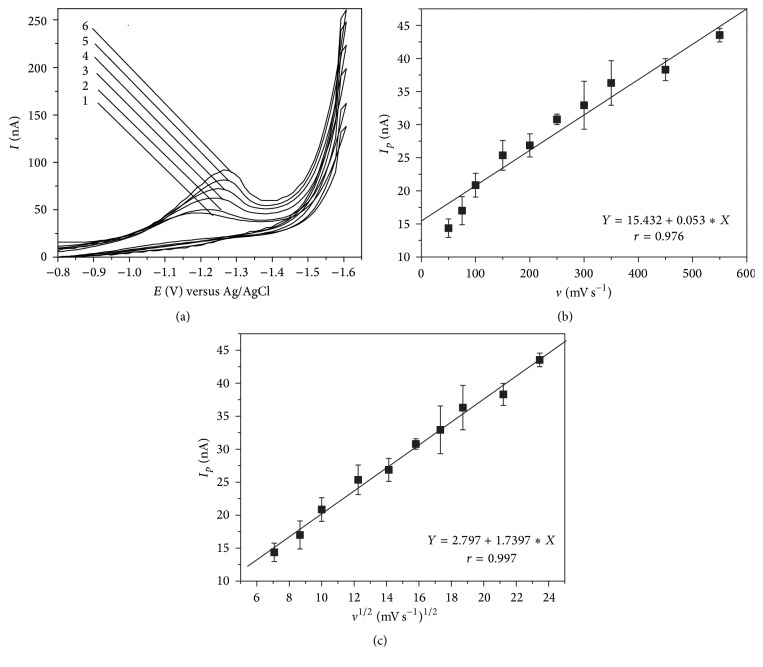
Study of the electrochemical process of EE2 (119.5 *μ*g L^−1^) in Britton-Robinson buffer (pH 7.0) at HMDE by cyclic voltammetry. (a) Cyclic voltammograms with scan rates: (1) 50 mV s^−1^; (2) 100 mV s^−1^; (3) 200 mV s^−1^; (4) 300 mV s^−1^; (5) 450 mV s^−1^; (6) 550 mV s^−1^. (b) Dependence of the peak current intensity as function of scan rate. (c) Dependence of the peak current intensity as function of square root of the scan rate.

The electrochemical behavior of EE2 in pH 7.0 B-R buffer was investigated by cyclic voltammetry at different scan rates at an HMDE (Figure 2(a)). An irreversible reduction peak is observed at −1.24 ± 0.03 V, which is attributed to the two-electron reduction of the 17-ethinyl group (-C≡CH) to form the 17-vinyl-estradiol (-CH=CH2). The peak potential shifts to more negative values increasing scan rate indicating an irreversible process [10, 22]. The choice of differential pulse adsorptive cathodic stripping voltammetry was predicated on the irreversible nature of EE2 reduction at the HMDE. Figure 2(a) shows that the EE2 peak current, *I*_*p*_, increases with increasing scan rate. Figures 2(b) and 2(c) show the dependence of *I*_*p*_ on scan rate and the square root of scan rate, respectively.  Figure 2(b) indicates that the relationship between peak current and scan rate is not linear and has a correlation coefficient equal to 0.976. This suggests diffusion control of the electrochemical reaction as indicated by the linearity (*r* = 0.997) of the plot of peak current versus the square root of scan rate in Figure 2(c). A plot of log⁡*I*_*p*_ versus the log of scan rate also was prepared and exhibited a linear correlation (*r* = 0.997) governed by the equation log⁡*I*_*p*_ = 0.390 + 0.456 · log⁡*v*, where *v* is the scan rate in mV s^−1^. The slope of the equation (0.456) is very close to 0.5, which is the value for a diffusion controlled electrochemical process.

### 3.2. Optimization of the Analytical Procedure

Voltammetric measurements of EE2 using DP AdCSV were independent of pH in the range pH = 6.0–9.0. For example, the peak current of EE2 was 1.6 ± 0.18 and 1.9 ± 0.2 nA at pH 6.0 and 9.0, respectively. In all cases, well-defined peaks were observed. These results indicate that the method can be applied to different natural water samples, which may have pH values ranging from slightly acidic (6.0) to slightly alkaline (8.0, seawater). pH 7.0 was chosen for subsequent studies.

**Figure 3:**
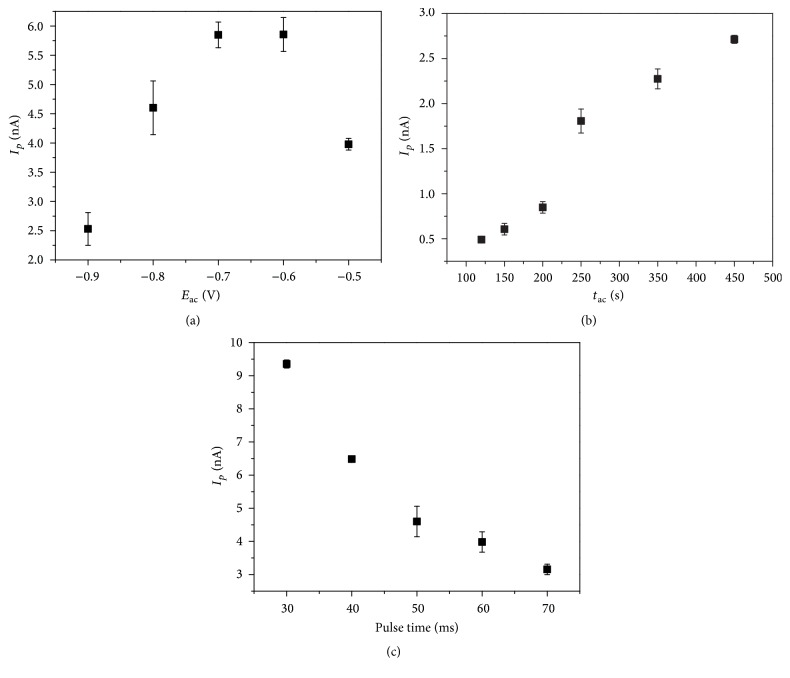
Optimization of the voltammetric parameters for the determination of EE2 (20.0 *μ*g L^−1^) by DP AdCSV. (a) Effect of accumulation potential with scan rate: 90 mV s^−1^, equilibrium time: 5 s; *t*_ac_: 150 s; pulse amplitude: 70 mV; and pulse time: 50 ms. (b) Effect of accumulation time with scan rate: 60 mV s^−1^; equilibrium time: 5 s; pulse amplitude: 50 mV; pulse time: 40 ms; and *E*_ac_: −0.7 V. (c) Effect of pulse time with scan rate: 60 mV s^−1^; equilibrium time: 5 s; *t*_ac_: 150 s; pulse amplitude: 50 mV; and *E*_ac_: −0.7 V.

Optimization of the voltammetric parameters used for DP AdsCSV indicated that some parameters significantly influenced the analytical response (Figure 3) but that scan rate, pulse amplitude, and equilibrium time did not (data not shown). The equilibrium time did not influence the peak current, and 5 s was chosen. Based on the scan rate dependence of *I*_*p*_, a value of 60 mV s^−1^ was chosen; scan rates of 45 to 60 mV s^−1^ showed a well-defined peak. The pulse amplitude did not influence the peak current, and a value of 50 mV, which produced a well-defined peak, was chosen.

Figure 3(a) shows the influence of the accumulation potential (*E*_ac_) on the peak current of EE2 from −0.9 to −0.5 V. The smallest peak current is observed at −0.9 V; the highest current of about 5.8 nA is observed at −0.7 and −0.6 V. An accumulation potential of −0.7 V was chosen, because of the lower standard deviation of the signal. The effects of accumulation time (*t*_ac_) on the analytical signal are demonstrated in Figure 3(b). The peak current increases as a function of preconcentration time. A duration of 120–150 s was chosen for subsequent studies, but longer times can be used to determine trace concentrations of EE2 in environmental samples without saturating the electrode surface.  Figure 3(c) shows the results obtained upon optimizing the pulse time. The peak current decreases with increasing pulse time, as expected. However, this parameter also influences the resolution of the voltammograms. Thus, a pulse time of 40 ms, which gives a well-defined peak and a reasonable peak current, was chosen.

From the above results, an accumulation potential of −0.7 V, an accumulation time of 150 s, a scan rate of 60 mV s^−1^, an equilibrium time of 5 s, a pulse time of 40 ms, a pulse amplitude of 50 mV, an HMDE area of 0.32 mm^2^, a potential sweep of −0.95 to −1.4 V, and a pH 7.0 B-R buffer were selected as optimal conditions for the determination of EE2 by DP AdsCV. These conditions are consistent with the literature [10]. For example, Ghoneim et al. developed a method for determining EE2 in pharmaceutical formulations and human plasma at a mercury electrode using square-wave adsorptive cathodic stripping voltammetry. The parameters were an accumulation potential of −0.8 V, an accumulation time of 6–70 s, a scan rate of 1.2 V s^−1^, an equilibration time of 5 s, an amplitude of 70 mV, an HMDE area of 0.026 cm^2^, and a pH 7.0 B-R buffer [10].

### 3.3. In-House Validation of the Procedure

**Figure 4:**
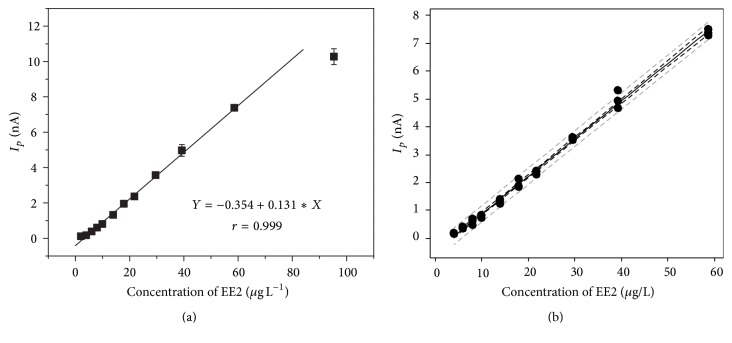
Calibration and linearity studies for quantification of EE2 by DP AdCSV. (a) Standard addition curve for EE2 with B-R buffer (pH 7). (b) Analytical curve (—) with confidence intervals (CI) (—  —) and prediction intervals (PI) (–—) at 95% confidence level.

Quantification of EE2 by DP AdCSV in the present study was based on the standard addition method. Linearity was tested over a concentration range of 2.0 to 96.0 *μ*g L^−1^ using a 150 s accumulation time.  Figure 4(a) shows that the peak current for EE2 reduction increases linearly with concentration up to 60.0 *μ*g L^−1^. Above 60.0 *μ*g L^−1^, *I*_*p*_ does not increase linearly with hormone concentration. Thus, data obtained from 4.0 to 60.0 *μ*g L^−1^ were used to establish the linear regression and to determine the coefficient (*R* ^2^) of adequacy-of-fit.  Figure 4(b) shows the plot of peak current versus concentration with confidence intervals (CI) and prediction intervals (PI) for the linear range studied. The peak current replicates in Figure 4(b) were generally within the predicted range at the 95% confidence level. However, one point (about 40.0 *μ*g L^−1^) showed a greater deviation from the mean current value and could be an outlier. The Grubbs test at the 95% confidence level [19, 20], which is based on the difference between the mean and extreme values considering the standard deviation, was applied. The value of *G*_obtained_ (1.07) was lower than 1.15, which indicates that the tested value is not an outlier and cannot be rejected from the analytical curve. The resulting standard addition curve indicates that the method shows good linearity (*R* ^2^ = 96%) up to 60.0 *μ*g L^−1^ and follows the relationship *I*_*p*_ (nA) = 0.131 ± 0.001 × *C*_EE2_ (*μ*g L^−1^) − 0.354 ± 0.009 (*n* = 3). Linear regression was conducted by ANOVA at the 95% confidence level. *F*_regression_ was 7,702.26, and *F*_critical_ was 4.196 (*p* < 0.05), which establishes the statistical significance of the fitted curve. The adequacy-of-fit of the curve was tested by the lack-of-fit procedure of linear regression (*F*_lof_) at the 95% confidence level. The result *F*_lof_ (1.31) < *F*_critical_ (2.447) for *p* > 0.05 shows that there was no lack-of-fit in the linear model constructed.

The values of LOD and LOQ determined in this work were 0.49 and 1.63 *μ*g L^−1^, respectively, for EE2 (accumulation time: 150 s). These limits are similar to those reported in the literature using electrochemical methods [9, 10]. The LOQ may not be adequate for EE2 quantification in natural water using a 3 min accumulation time, but an improved LOQ can be obtained with a longer accumulation period (Figure 2(b)). The linear working range was restricted to 1.63 to 60.0 *μ*g L^−1^ in this work. Quantifications below the LOQ are not reliable using a 150 s accumulation time.

The precision of the method was evaluated in terms of the repeatability (Re) and intermediate precision (IP). Values of 9.2 and 13.0% were obtained for Re and IP, respectively. The RSD values obtained were below 20% and are considered to be suitable [21, 23, 24].

The selectivity of EE2 analysis was examined in the presence of organic matter such as humic acid (HA). HA significantly interfered with the determination of EE2 by DP AdCSV over the entire range studied. The interference consisted of a shift of the peak for EE2 reduction (−1.163 ± 0.001 V) to a more positive potential (−1.124 ± 0.006 V). HA can coadsorb with the hormone on a mercury electrode [12]. The interference was minimized by dilution and filtration of the sample before measurement. Elimination of the interference was established by accuracy tests, which were carried out with real samples from the Guarapuava River, state of Paraná (Brazil).

Table 1 shows the results of the accuracy test of the method proposed for aqueous solutions and natural waters. Freshwater reference material with certified concentrations of pharmaceuticals was not available; thus, accuracy was evaluated by recovery tests. Recoveries ranged from 94 to 103% for both samples, indicating that the proposed procedure is accurate. The tolerance of recovery tests in trace analysis is about ±20% [19, 24, 25].

## 4. Conclusions

This carefully validated work describes the first procedure for determining EE2 by voltammetry. The results indicate that hormone screening can be performed without the need for sample pretreatment (extraction or clean-up). Thus, voltammetry at a mercury electrode is a viable procedure for analyzing organic compounds at trace levels. EE2 can be quantified with good accuracy and precision in aqueous samples using a HMDE. Voltammetric measurements are advantageous in monitoring studies (screening), and chromatography can be used to confirm the presence of the hormone in samples exhibiting a positive voltammetric response.

## Figures and Tables

**Table 1:** Validation tests for accuracy of the method in water.

Sample	Concentration of EE2 (*μ*g L^−1^)		Recovery (%)
	Spiked	Determined	
Aqueous solutions	7.8	8.0 ± 1.0	102.5
Aqueous solutions	10.0	10.0 ± 1.3	100.0
Natural water	16.0	15.0 ± 1.2	93.7
